# Selective Apheresis of C-Reactive Protein for Treatment of Indications with Elevated CRP Concentrations

**DOI:** 10.3390/jcm9092947

**Published:** 2020-09-12

**Authors:** Stefan Kayser, Patrizia Brunner, Katharina Althaus, Johannes Dorst, Ahmed Sheriff

**Affiliations:** 1 Pentracor GmbH, 16761 Hennigsdorf, Germany; kayser@pentracor.de; 2 iAdsorb GmbH, 10787 Berlin, Germany; patrizia.brunner@gmx.de; 3 Department of Neurology, University of Ulm, 89081 Ulm, Germany; katharina.althaus@uni-ulm.de (K.A.); johannes.dorst@uni-ulm.de (J.D.); 4 Medizinische Klinik m.S. Gastroenterologie/Infektiologie/Rheumatologie, Charité Universitätsmedizin, 12203 Berlin, Germany

**Keywords:** CRP, apheresis, stroke, inflammation

## Abstract

Almost every kind of inflammation in the human body is accompanied by rising C-reactive protein (CRP) concentrations. This can include bacterial and viral infection, chronic inflammation and so-called sterile inflammation triggered by (internal) acute tissue injury. CRP is part of the ancient humoral immune response and secreted into the circulation by the liver upon respective stimuli. Its main immunological functions are the opsonization of biological particles (bacteria and dead or dying cells) for their clearance by macrophages and the activation of the classical complement pathway. This not only helps to eliminate pathogens and dead cells, which is very useful in any case, but unfortunately also to remove only slightly damaged or inactive human cells that may potentially regenerate with more CRP-free time. CRP action severely aggravates the extent of tissue damage during the acute phase response after an acute injury and therefore negatively affects clinical outcome. CRP is therefore a promising therapeutic target to rescue energy-deprived tissue either caused by ischemic injury (e.g., myocardial infarction and stroke) or by an overcompensating immune reaction occurring in acute inflammation (e.g., pancreatitis) or systemic inflammatory response syndrome (SIRS; e.g., after transplantation or surgery). Selective CRP apheresis can remove circulating CRP safely and efficiently. We explain the pathophysiological reasoning behind therapeutic CRP apheresis and summarize the broad span of indications in which its application could be beneficial with a focus on ischemic stroke as well as the results of this therapeutic approach after myocardial infarction.

## 1. General Introduction

Inflammatory processes involve a plethora of signaling pathways and affect the whole body, even if their origin is most often locally restricted in an acute setting. Mounting an inflammatory response is the body’s strategy to primarily eliminate any cause of tissue damage and subsequently repair the injury [1]. This is rooted in the evolutionary background that damage is mainly caused by pathogens or at least exacerbated by them within an external wound. In this case the elicited inflammation is beneficial in fighting infiltrating bacteria or viruses as well as restoring tissue homeostasis. However, healing of injured tissue often happens at the cost of still healthy tissue/cells and involves additional cell death as collateral damage [2]. In specific situations, these negative effects outweigh the positive aspects of the inflammatory reaction. Whenever an injury is “sterile”, meaning it occurred internally without pathogen involvement, inflammation aggravates deterioration by elimination of additional cells, which were either vital or only slightly and reversibly impaired. This happens for example after ischemic injury like stroke or myocardial infarction, leading to a larger extent of organ damage, increased scarring and thereby worsening clinical outcome [3,4]. Likewise, negative effects dominate in situations when the immune system produces an excessive general reaction that is not justified by the trigger [5]. For example, during acute pancreatitis, a systemic inflammatory response syndrome (SIRS), or an acute bacterial or viral infection (Sepsis) the inflammation might cause widespread tissue injury, which might result in multiple organ failure [6].

Although a plentitude of proteins is involved in inflammation, many of them are cytokines or modulators that do not actively participate in the elimination of pathogens or cells [1]. Several mediator proteins play a key role.

One of the acute-phase mediators directly involved in these pro-inflammatory processes is C-reactive protein (CRP) which was discovered by Tillett and Francis in 1930 [7]. CRP is well-established as one of the most reliable markers of inflammation, rising dramatically during any type of inflammation. It has been shown that CRP as an inflammatory mediator not only reflects tissue damage, but also aggravates the severity of damage and contributes causally to course and outcome of various diseases [8]. Therefore, CRP has to be regarded not only as a marker, but also as an active pro-inflammatory protein.

## 2. Role of CRP

CRP is a sensitive, reliable and early indicator of inflammation and infection. Evolutionarily highly conserved, this pentameric molecule is part of the ancient humoral immune response and involved in various immunological pathways as a key mediator [9,10]. It is predominantly synthesized and secreted into the blood circulation by hepatic cells as a response to trauma, inflammation, or infection. In these situations, the proinflammatory cytokines interleukin 6 (IL-6) and, to a lesser extent, interleukin 1β (IL-1β) as well as tumor necrosis factor @(TNF@) induce CRP expression on the transcriptional level [11,12,13,14]. Following an acute phase stimulus, serum CRP values increase up to levels a few thousand times higher than the normal (healthy) concentration of human CRP (0.05 to 3000 mg/L) [15,16]. The half-life in plasma is about 19 h [17,18].

After secretion, CRP efficiently detects and opsonizes bacteria upon their infiltration and initiates their phagocytosis by activation of complement [19,20]. This is probably its original purpose as one of the most ancient proteins within the humoral immune system.

**Figure 1:**
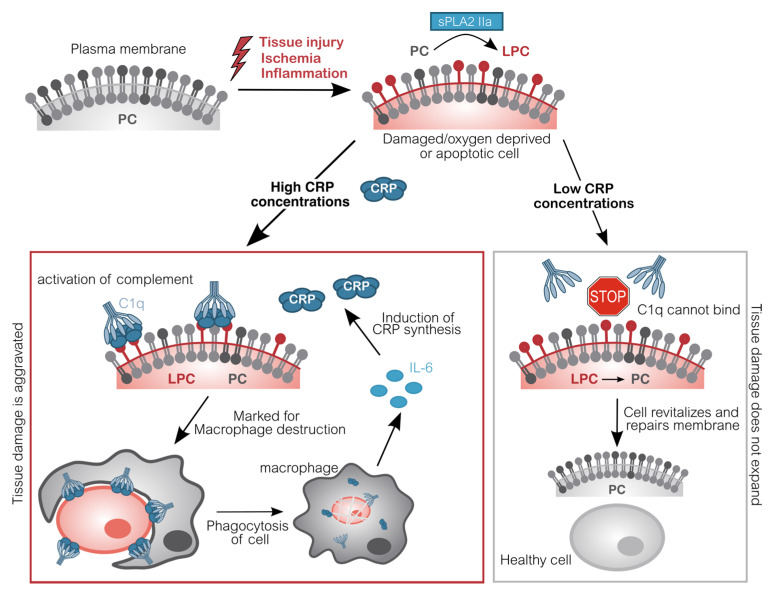
Molecular pathomechanism of CRP-mediated tissue damage. Upon inflammation or acute oxygen-deprivation, cells display a dramatic shortage of adenosine triphosphate (ATP). ATP is essential to prevent apoptosis which manifests in the outer cell membrane: Phosphatidylcholine (PC) is converted into lyso-phosphatidylcholine (LPC) by phospholipase (sPLA2 IIa). Due to the lack of ATP, this alteration cannot be reversed. CRP subsequently binds to LPC on anaerobic cells and recruits complement factors (C1q-C4). These opsonized cells will be disposed by phagocytes, which in turn induce CRP synthesis. Without CRP or in situations with low CRP concentrations (e.g., after CRP apheresis), energy deprived-cells are spared and may switch back to aerobic metabolism, repair molecular changes and revitalize again, leading to an overall reduced tissue damage [41,43,44,45]. CRP C-reactive protein; C1q Complement component 1q; IL-6 Interleukin 6; LPC Lysophosphatidylcholine; PC Phosphatidylcholine; sPLA2 IIa secretory phospholipase A2 type IIa.

However, CRP also detects and binds to endogenous cells [21,22]. Cells, which are either apoptotic, energy-depleted, or simply exposed to stressors like the acidic and often ischemic environment of inflammation display conformational and biochemical changes of their membrane [23]. One of these changes is the formation of lyso-phosphatidylcholine (LPC) by partial hydrolyzation of phosphatidylcholine (PC). To this end, one of its two fatty acid groups is removed by the secretory phospholipase A2 type IIa (sPLA2 IIa) [24,25]. This phospholipase is secreted and activated by inflammation (IL-6) and marks the beginning of detrimental destruction of still viable tissue after e.g., ischemia [26,27,28,29]. LPC is thereby accessible in the plasma membrane of dead, damaged, or inflamed cells. The CRP pentamer binds to LPC with high avidity in a so-called cooperative manner and subsequently mediates the elimination of these cells, similarly to infiltrating pathogens, by activating the classical complement pathway [30,31,32,33,34,35]. Complement C1q binds to CRP directly and mediates the binding of C2–C4 [36]. Thus, these cells are irreversibly marked for phagocytes which dispose the marked cells. Phagocytes in turn secrete IL-6 which induces the synthesis of additional CRP by the liver, subsequently amplifying the immune response. This way, more cells become marked by CRP (Figure 1).

Importantly, this mechanism facilitates binding of CRP to actually still vital cells, which may potentially regenerate with more CRP-free time. By interacting with complement, CRP triggers the destruction and therefore negatively affects the regeneration of tissue. By now, a large body of data obtained from animal experiments demonstrates that this CRP-mediated mechanism plays an active role in exacerbating ischemia and reperfusion-induced damage [37,38,39,40,41,42,43].

On the molecular level it is not fully elucidated yet whether pro-inflammatory signaling is mediated by the pentameric, native form of CRP, or if CRP dissociates into its non-covalently bound monomers upon binding to LPC, which then exhibit novel binding capacities and other specific functions [46,47,48]. Publications which described anti-inflammatory actions of pentameric CRP hypothesized that CRP switches functions by undergoing structural changes. Although various quaternary structures of CRP are still not well proven in the physiological context, it might well be possible that CRP monomers exist in specific inflammatory microenvironments and represent different stages of inflammation [47,49]. It has been clearly shown that CRP is secreted in its native, pentameric form by the liver and-if at all-only dissociates locally within inflamed tissue. Hence, therapeutic interventions are more efficient targeting pentameric CRP as high circulating levels are the actual source for its detrimental action [50,51]. Its known physiological function is the disposal of cells (bacteria, necrotic and apoptotic cells).

To date, no pharmacologic inhibitor of inflammation has been proven to be successful in ischemia-related injuries, since they all featured unfavorable pharmacokinetic profiles or serious side effects. Therefore, a different strategy is needed to target the detrimental inflammatory response [43,52]. Specifically, targeting avoidable organ damage caused by the action of CRP represents a promising therapeutic option [43,53]. Decreasing CRP levels could potentially protect salvageable cells and give them more time to recover. Therefore, removing CRP from the blood circulation interrupts the innate cascade and reduces tissue damage [44]. Accordingly, CRP apheresis may potentially present a promising, highly efficient, and well-tolerated therapeutic option.

## 3. CRP Apheresis

Extracorporeal apheresis refers to the physical removal of substances from the blood by means of filtration, precipitation or adsorption. Immunoadsorption defines the specific binding of an immunologic protein by an adsorber matrix. The elimination of pathogenic substances from the blood in extracorporeal apheresis constitutes an established therapeutic measure in the clinical routine of numerous diseases.

**Figure 2:**
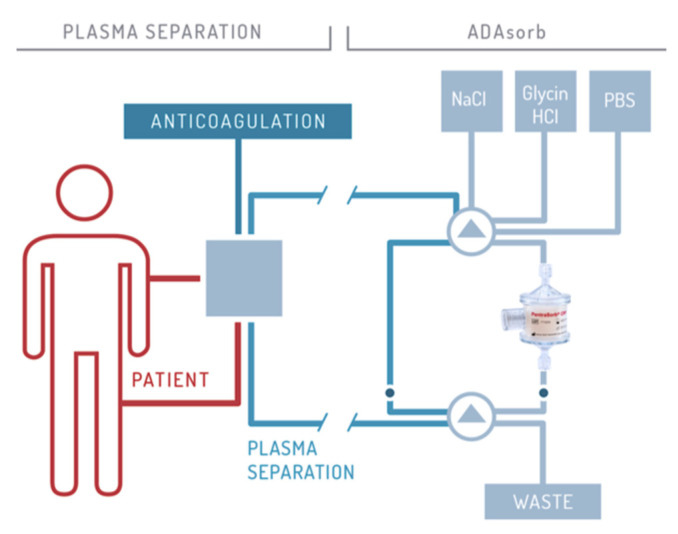
Schematic illustration of CRP apheresis. The procedure is described in detail by Ries et al. 2019 [45].

The CRP adsorber system (PentraSorb^®^ CRP, Pentracor GmbH, Hennigsdorf, Germany) features an agarose-based resin, which contains a phosphocholine-derivative as ligand for CRP and is thereby capable of selectively depleting CRP from blood plasma with an efficiency of up to 94% (under laboratory conditions) [54]. The adsorber is regenerable and can be used up to a maximum cumulative treatment time of 24 h (contact with human plasma ≤24 h, according to CE license). In between treatments the adsorber has to be stored in sodium azide at 2–8 °C. CRP apheresis is executed in cycles, alternating between loading of the adsorber with plasma and regeneration of the column, that follows a fixed sequence of washing solutions. Loading and washing are controlled by a software module for automatic plasma flow management (ADAsorb, medicap clinic GmbH, Ulrichstein, Germany; Figure 2). Blood can be drawn via central or peripheral venous access (cubital veins). Plasma separation is performed by a blood centrifuge and blood is anti-coagulated 1:15 with citrate buffer (ACD-A; 3% citrate) or heparin. The usual plasma flow through the adsorber is between 25 to 35 mL/min. Blood flow ranges between 40 and 65 mL/min.

During one treatment, 6000 mL of plasma are usually processed in 12 cycles. A continuous monitoring of vital parameters, blood pressure and heart rate has to be carried out. Processing of 6000 mL blood plasma takes 4–5 h, depending on the blood flow. Patients can be treated an infinite amount of times with CRP apheresis, as the blood loss is only minimal. Depending on CRP level and indication, two to ten treatments on consecutive days are performed. So far, no side-effects have been reported [45,55,56,57].

The main advantages of CRP apheresis are the selective removal of the agent by the highly specific ligand and the good controllability of the process, since unlimited plasma volumes can be processed to achieve the desired CRP reduction. Drugs are not removed by CRP apheresis.

## 4. CRP Apheresis after Ischemic Tissue Damage

The extent of tissue damage during and after an acute traumatic incident defines outcome and follow-up health. Ischemic lesions, predominantly acute myocardial infarction (AMI) and ischemic stroke, generate initial organ damage in the acute zone by cell death due to oxygen deprivation and its magnitude is primarily determined by its duration [58]. Further, neighboring cells which are deprived of oxygen for a shorter duration or to a lesser extent are damaged but salvageable and constitute the area at risk (AMI) or penumbra (stroke) [58,59]. The first line of therapy constitutes the restoration of blood flow to limit the initial ischemic injury. This reperfusion, even though essential to decrease mortality and morbidity, is attended by an intense and maladaptive immune response, which augments and accelerates the organ damage and includes the still viable but damaged tissue [60,61]. The elimination of salvageable cells by CRP through this mechanism mediates reperfusion injury and critically contributes to the already existing deterioration [62,63]. CRP apheresis aims to remove circulating CRP after AMI and ischemic stroke in order to reduce acute tissue injury and ischemic reperfusion injury.

### 4.1. Myocardial Infarction

Patients who recover from AMI often suffer from reduced quality of life and very high risk of severe complications later on (e.g., second infarct), which implies a huge burden for the health system. This risk correlates significantly with the extent of myocardial injury and scarring [64,65].

It has long been established that inflammation especially mediated by the innate immune system extends myocardial injury, however, anti-inflammatory strategies to minimize myocardial necrosis have failed so far, maybe because these processes are also needed for healing and cardiac repair [3,4,52,66]. While baseline CRP levels in the healthy state are established as predictor of the incidence of cardiovascular disease [67,68,69], serum CRP concentration during and after AMI correlates with clinical outcome [16,17,70,71,72,73,74]. It is well known that high peak CRP levels during the acute phase response after AMI correlate with larger infarct size and higher mortality as well as incidence of major adverse events [17,74,75]. This has been described for more than two decades now and is in line with the described pathological function of CRP, eliminating cells in the area at risk [8,23,76]. This area contains cells, which could partially recover after revascularization and reperfusion, but are finally destroyed by immune-mediated mechanisms, as explained above and shown in detail in numerous experimental approaches focusing specifically on AMI [39,40,63,70,77,78]. Targeting CRP in AMI has therefore been proposed previously, but was never achieved due to non-functioning therapeutic approaches [43,79,80,81].

**Figure 3:**
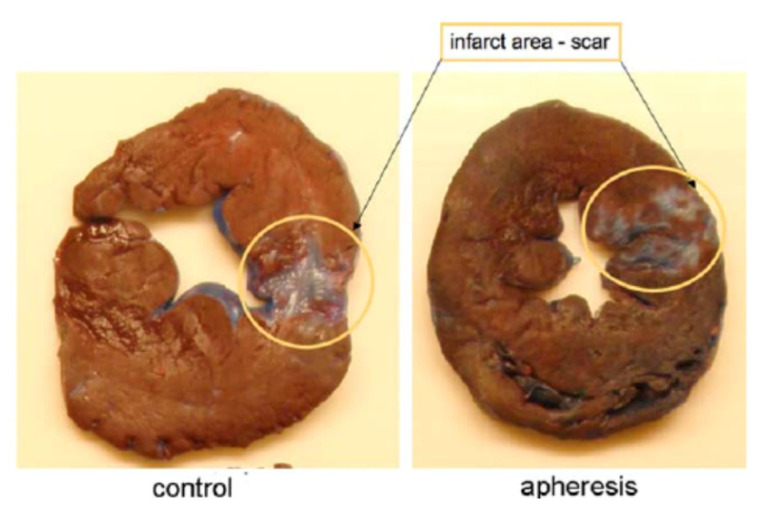
Porcine Heart Slices after AMI with and without CRP apheresis. Slices of the left ventricle 14 days after AMI. Slices were generated after an Evans Blue staining of the heart. Circles localize a characteristic transmural scar of a control animals (left) versus spotted scar morphology after CRP apheresis (right). Figure previously published and taken from [41].

Preclinical studies on the efficacy of specific extracorporeal depletion of CRP have been successfully performed in a porcine animal model of AMI [41,42]. In this study, a mean reduction of CRP levels by about 50%, a significant reduction of the infarct size and a stabilization of the ejection fraction was observed. Interestingly, a completely different scar morphology was detected in animals after CRP apheresis compared to controls [41]. A smaller scar tissue and more vital heart muscle reflected the efficacy of this treatment strategy (Figure 3, previously published and taken from [41]). AMI was therefore selected as indication for the first clinical trial of CRP apheresis. CRP apheresis was applied in patients with ST-elevation myocardial infarction (STEMI) (CAMI-1 trial: “Selective depletion of C-reactive protein by therapeutic apheresis (CRP apheresis) in acute myocardial infarction”, DRKS ID: DRKS00008988). Just recently, this multi-center clinical trial has been finished and first data were shown in Case reports and a publication describing 13 patients as a preliminary report [44,45,55,56].

The CAMI-1 trial tested the hypothesis whether specific depletion of CRP by CRP apheresis can reduce myocardial infarct size in humans. Endpoints were safety, myocardial infarct size and function as well as CRP concentration in patients with acute STEMI. A total of 83 patients were recruited at 8 study centers. Plasma CRP levels were reduced by approximately 60% over all performed apheresis procedures in the CAMI-1 trial. Treatments were safe and well tolerated. There were no serious adverse effects associated with the treatment [45]. The magnitude of increase of CRP concentration during the acute phase response after STEMI correlated significantly with the infarct size in control patients. Patients with similar initial CRP increase, who subsequently underwent CRP apheresis, showed smaller infarct sizes as well as improved left ventricular function and wall motion (strains) compared to control patients (*unpublished data-submitted*). Currently, a CAMI-1 registry is on-going, collecting more data (DRKS00017481) [44].

### 4.2. Ischemic Stroke

Stroke is the third most frequent cause of death and the leading cause of serious, long-term disability worldwide. This disease has a tremendous personal, familiar and socioeconomic impact. More than 80% of patients suffer from ischemic stroke [82]. To date, restoring rapid reperfusion of the brain constitutes the only established therapeutic strategy to reduce the size of the infarct and the consequences of the disease [83]. However, similar mechanisms to AMI take place and inflammation plays an important role in various stages of ischemic stroke, because several humoral and cellular mechanisms are set in motion by the occlusion and subsequent therapeutic reperfusion [84,85]. These mechanisms may explain why some patients with ischemic stroke suffer from severe neurological symptoms despite early and successful recanalization. Several findings substantiate the hypothesis that CRP plays a similar pathological role as shown in AMI, facilitating the elimination of energetically challenged and compromised cells in the penumbra.

First, various publications have shown an association between the early inflammatory response after ischemic stroke and the clinical outcome. The early inflammatory response after stroke has been identified as a key prognostic factor [86,87]. Patients with favorable clinical outcome feature significantly lower levels of inflammatory parameters, especially CRP, compared to patients with poor outcome. Previous studies have described an association between high CRP values after acute stroke and negative prognosis [88,89,90,91]. Muir et al. have shown that CRP levels measured within 72 h after stroke predict mortality over an observation period of up to 4 years [92]. According to Winbek et al., CRP levels 24 and 48 h after onset of symptoms affect prognosis, but not their concentration at admission [87]. In another stroke study, patients who died during the study period had significantly higher CRP levels at admission compared to survivors and CRP levels correlated with the clinical outcome after 3 months follow-up [86]. Further, studies in a rat animal model have shown that infusion of human CRP enlarges cerebral infarct areas after acute occlusion via a complement-dependent mechanism [37].

Based on this background, a clinical trial investigating selective CRP apheresis after ischemic stroke was initiated (CASTRO1 trial: “Selective Depletion of C-reactive Protein by Therapeutic Apheresis (CRP-apheresis) in Ischemic Stroke”, ID: NCT0441723). The CASTRO trial is designed as a randomized, controlled, multicentric interventional pilot trial. The aim of the CASTRO trial is to evaluate if CRP apheresis can be applied safely in patients with ischemic stroke and efficiently lower the CRP level. Therefore, the primary endpoint is the type and frequency of adverse events and serious adverse events after apheresis. In addition, potential effects of CRP apheresis on clinical outcome parameters (cognitive measures, infarct volume, laboratory parameters) will be investigated.

Participants for this trial need to have an ischemic stroke with or without intravenous lysis and recanalization therapy. The National Institutes of Health Stroke Scale (NIHSS) has to be between 1–24 in order to exclude patients with severe, potentially complicated disease courses. CRP needs to increase ≥5 mg/L within 72 h after the incident and/or serum CRP concentration needs to be larger than 10 mg/L. We aim to include 20 patients which are 1:1 randomly assigned to either the control group (standard guideline therapy) or CRP apheresis in addition to the standard guideline therapy. The standard therapy of acute ischemic stroke is carried out according to the guidelines of the European Academy of Neurology [93].

**Figure 4:**
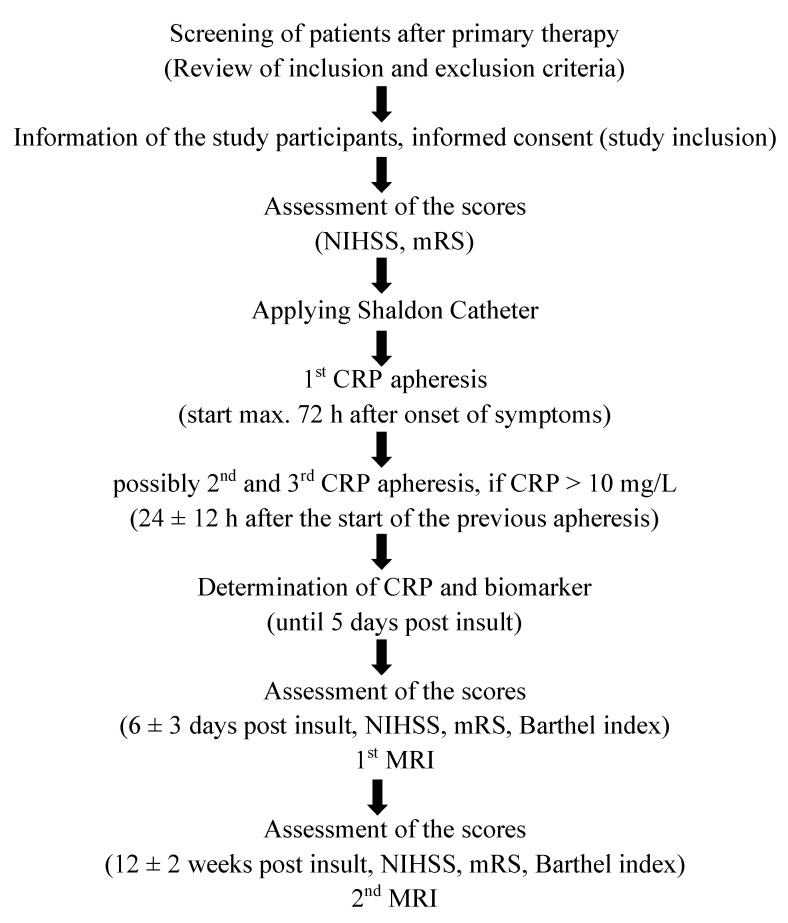
Study flow of the CASTRO1 trial. MRI magnetic resonance imaging; NIHSS National Institute of Health Stroke Scale; mRs modified Rankin scale.

Exclusion criteria are severe dysphagia (risk of aspiration pneumonia), clinical or laboratory evidence of systemic infection, contraindications against apheresis, Modified Rankin Scale (mRS) before index event ≥ 3, intracranial hemorrhage, epileptic seizure in the context of the acute event, pregnancy, and lactation. Treatment and study regime will be implemented into the clinical standard diagnostic and therapeutic regime after stroke. Since CRP levels begin to rise approximately 8 h after the ischemic incident and reach their peak after 24 h, the first CRP apheresis will be carried out within 72 h after onset of symptoms. Therefore, CRP apheresis will not delay acute guideline therapies of stroke, such as intravenous lysis and intraarterial thrombectomy. The complete study flow is illustrated in Figure 4.

To investigate whether CRP apheresis improves clinical outcome parameters after ischemic stroke, patients will undergo assessments according to standardized clinical scales, namely National Institute of Health Stroke Scale (NIHSS) score, Barthel ADL index (BI), modified Rankin scale (mRS) and measurements of infarct volume (via magnetic resonance imaging; MRI). In addition, immunological and neurodegenerative biomarkers (interleukin-6, serum amyloid A) will be evaluated to objectify a potential beneficial effect of CRP apheresis on inflammatory pathways. Measurements of primary and secondary outcome parameters will be performed at baseline (before first apheresis), daily during apheresis, and 90 days after stroke.

Immunoadsorption with the PentraSorb^®^ CRP is performed with the ADAsorb apheresis device as described in detail in 3. CRP apheresis is performed for a maximum amount of three times (three days) or until CRP concentration is below 10 mg/L.

## 5. CRP Apheresis in Other Indications

Both AMI and ischemic stroke feature a common underlying pathophysiology and the therapeutic application and benefit after AMI has been already shown. However, reduction of dramatically high CRP concentrations in other indications which are not defined by an ischemic pathophysiology could also be beneficial. The overcompensating immune reaction which often triggers SIRS after surgery, causes detrimental deterioration during acute pancreatitis, or mediates a cytokine storm after infection, could be dampened with CRP apheresis. Therefore, clinical trials investigating the safety and efficacy of CRP apheresis during pancreatitis and after coronary bypass surgery are ongoing (CAPRI1-study DRKS00014265; CABY1-study DRKS00013012). Further, first patients suffering from Covid-19 have been treated with CRP apheresis in order to inhibit the CRP-mediated autoimmune response leading to respiratory failure and multi-organ failure [57,94].

## 6. Conclusions and Outlook

CRP has been established as a general biomarker of inflammation and infection in clinical practice. Recently, its role as a stable and highly useful prognostic factor for cardiovascular and cerebral disease in healthy individuals has been widely acknowledged and utilized [95,96]. However, the characterization of CRP as not only a biomarker but also a mediator or even trigger of immunological destruction of tissue is widely ignored [8,37,39].

Therapeutic CRP removal by immunoadsorption might present a logical and promising therapy for pathologies in which the extent of tissue damage is aggravated by inflammation and correlated with a worse clinical outcome, including ischemic events.

CRP apheresis has been applied successfully in a controlled multi-center trial in patients with myocardial infarction (CAMI-1 trial). It showed very few and only moderate side effects and managed to significantly reduce CRP levels, thereby positively affecting infarct size and left ventricular ejection fraction [44,45,55,56]. Applying CRP apheresis in ischemic stroke is the next plausible step. However, the immunological situation in the brain is different. Neurons have a low tolerance to oxidative stress, and the physiologically important blood-brain-barrier may impair the effectiveness of this method [97,98,99].

The CASTRO study will show whether CRP apheresis can be safely performed in patients with ischemic stroke and also provide preliminary results whether reducing the concentration of serum CRP levels facilitates reduction of tissue damage of the brain, consequently improving clinical outcome measures compared to the control group.

Other anti-inflammatory therapies have been investigated in AMI and ischemic stroke, such as colchicine [100], anti-CD18 agents [101] and agents targeting IL-1 or IL-6 [102,103,104]. CRP removal intends to stop the destruction of tissue already during the acute event. Furthermore, targeting specifically and selectively CRP may constitute a superior choice because it does not cause a pleiotropic effect. The maximum removal of CRP in patients was 79% by now. This leaves enough CRP for potential repair processes. Importantly, cardiac or neural repair is not impaired by the intervention as opposed to former pharmacological interventions like the methylprednisolone trial in myocardial infarction which resulted in a catastrophic outcome [105].

Preliminary evidence suggests that CRP apheresis induces very few side effects and features a low risk profile [45]. One drawback is that the procedure takes relatively long. Nevertheless, CRP apheresis fits well into the management of stroke patients because it does not collide with acute measures and may therefore complement methods aiming at reperfusion.

The acute inflammatory response has two facets. For one thing it plays a key role in initial host defense against infections. But on the downside, it can cause collateral damage of tissues. Especially in situations with an inciting sterile stimulus, the cost-benefit ratio is unfavorable.

CRP as an ancient protein of the innate immune system physiologically disposes cells and reacts to almost every disturbance of tissue homeostasis. Therefore, the span of potential indications for CRP apheresis is broad, and the ongoing clinical trials will illuminate whether this therapy is beneficial in these specific indications.
